# Disentangling the biological mechanisms underlying the effects of physical exercise in major depressive disorder: a comprehensive systematic review of randomized controlled trials

**DOI:** 10.1017/S0033291725100743

**Published:** 2025-07-16

**Authors:** Rosana Carvalho Silva, Mattia Meattini, Giulia Perusi, Sara Carletto, Marco Bortolomasi, Massimo Gennarelli, Bernhard T. Baune, Alessandra Minelli

**Affiliations:** 1 Department of Molecular and Translational Medicine, University of Brescia, Brescia, Italy; 2 Genetics Unit, IRCCS Istituto Centro San Giovanni di Dio Fatebenefratelli, Brescia, Italy; 3 Department of Clinical and Biological Sciences, University of Torino, Turin, Italy; 4 Clinical Psychology Unit, A.O.U. Città della Salute e della Scienza di Torino, Torino, Italy; 5 Psychiatric Hospital “Villa Santa Chiara”, Verona, Italy; 6 Department of Psychiatry and Psychotherapy, University of Münster, Münster, Germany; 7 Department of Psychiatry, Melbourne Medical School, University of Melbourne, Melbourne, VIC, Australia; 8 The Florey Institute of Neuroscience and Mental Health, The University of Melbourne, Parkville, VIC, Australia

**Keywords:** biological effects, biomarkers, inflammatory system, major depressive disorder, monoaminergic system, neurotrophic system, physical exercise, randomized controlled trials, stress-response system, systematic review

## Abstract

**Background:**

Major depressive disorder (MDD) is a disabling psychiatric condition in which physical activity provides clinical benefits. While exercise effectively alleviates depressive symptoms, its biological mechanisms remain unclear.

**Methods:**

This systematic review investigated the neurobiological effects of physical exercise on biomarkers in adults with MDD through randomized controlled trials, including studies assessing exercise interventions and reporting data on their biological effects.

**Results:**

A total of 30 studies, including 2194 participants, were included, examining the effects of physical exercise on various biological systems in patients with MDD. Exercise interventions had mixed effects on inflammatory markers, including interleukins, C-reactive protein, and tumor necrosis factor-α, suggesting a potential but inconsistent anti-inflammatory role. Neurotrophic factors, such as brain-derived neurotrophic factor showed promise as biomarkers of treatment response, but their role in clinical improvements remained inconclusive. Findings for the stress-response system, including cortisol and monoaminergic systems, primarily involving serotonin and dopamine, were limited and variable. Exercise demonstrated potential benefits in reducing oxidative stress and enhancing β-endorphin levels, although these effects were not consistently observed.

**Conclusion:**

This systematic review adopted a broader perspective than prior studies, exploring less-studied biological systems and identifying several limitations in the included studies, including small sample sizes, varying methodologies, and a predominant focus on biochemical markers. Future research should prioritize larger, standardized trials and particularly employ omics approaches to better understand the biological mechanisms underlying the effects of exercise in MDD. The findings highlight the complexity of exercise’s biological effects and emphasize the need for further research to clarify its mechanisms.

## Introduction

Major depressive disorder (MDD) is a debilitating psychiatric condition, ranked as the sixth most disabling disorder globally, affecting approximately 350 million people worldwide and significantly contributing to morbidity and mortality (Global Burden of Disease, 2020; Walker, McGee, & Druss, 2015; World Health Organization, 2017). Its pathophysiology involves alterations in neurotrophic pathways, inflammation, immune responses, endocrine function, the monoaminergic system, and stress-related pathways such as the hypothalamic–pituitary–adrenal axis (Anderson & Maes, 2014; Berger et al., 2016; Chocyk et al., 2013; Maffioletti, Minelli, Tardito, & Gennarelli, 2020). Current pharmacological treatments primarily target these biomarkers, particularly within the monoaminergic system. However, they often fail to fully alleviate symptoms and are associated with side effects, including weight gain, cardiovascular issues, and sexual dysfunctions (Dodd et al., 2021). Consequently, non-pharmacological interventions are increasingly recognized for their role in treating depression symptoms.

Physical exercise is an effective non-pharmacological strategy for MDD treatment, symptom alleviation, and as an adjunct to enhance the efficacy of antidepressant medications and other therapies. Several systematic reviews and meta-analyses have demonstrated that physical exercise significantly reduces depression symptoms (Cooney et al., 2013; Josefsson, Lindwall, & Archer, 2014; Krogh et al., 2017; Schuch et al., 2016). The latest guidelines for MDD management recommend regular physical activity at low to moderate intensity (30–40 minutes per session, three to four times per week, for at least 9 weeks) as a primary treatment for mild depression and an adjunct treatment for moderate cases (Lam et al., 2024; National Institute for Health and Care Excellence, 2022; Zhou, Puder, & Fabiano, 2024). A meta-review demonstrated that physical activity significantly improves depressive symptoms, with effects comparable to those of antidepressants and psychotherapy, recommending moderate to vigorous aerobic exercise, ideally combined with resistance training, performed two to three times per week for a total of 150 minutes under professional supervision (Stubbs et al., 2018).

Despite robust evidence on the effectiveness, the neurobiological mechanisms underlying exercise’s antidepressant effects are not fully understood. Previous systematic reviews and meta-analyses indicate that physical activity induces sustained responses in hormones, neurotrophins, and inflammatory/immune processes (Feter, Alt, Dias, & Rombaldi, 2019; Schuch et al., 2014). Exercise is known to modulate depression-related biomarkers, including interleukins (IL) such as IL-6, a key component of the immune and inflammatory systems, and other molecular signatures associated with MDD pathophysiology (Chow et al., 2022; Schuch et al., 2014).

A recent systematic review and meta-analysis investigating the chronic effects of physical activity on biomarkers in MDD patients included 12 randomized controlled trials (RCTs) with 757 participants. The study found that exercise significantly increased circulating levels of brain-derived neurotrophic factor (BDNF) and kynurenine while reducing depression symptoms compared to the control groups (da Cunha, Feter, Alt, & Rombaldi, 2023). Improvements in circulating BDNF, kynurenine, and IL-6 levels were associated with reductions in depression symptoms (da Cunha et al., 2023). Another systematic review and meta-analysis examined the effects of antidepressant treatment, with or without physical exercise, on inflammatory biomarkers in MDD patients (Fernandes et al., 2022). The authors identified six trials investigating biomarkers, such as IL-1β, IL-6, serum cortisol, and C-reactive protein (CRP), reporting significant methodological heterogeneity and conflicting results (Fernandes, Scotti-Muzzi, & Soeiro-de-Souza, 2022).

Although evidence highlights the neurobiological effects of physical exercise in MDD, findings remain inconsistent and predominantly focus on inflammatory pathways, with limited data on other biological mechanisms. Comprehensive and updated analyses are necessary to better understand the specific mechanisms underlying the effects of various forms of physical exercise in MDD treatment.

This review aims to address these gaps by updating knowledge on the biological mechanisms of exercise in MDD and broadening the scope to encompass a wider range of biological systems. This study broadens the scope of previous analyses by exploring less-studied biological systems beyond the immune-inflammatory and neurotrophic pathways, including oxidative stress, monoaminergic, and stress-response mechanisms. It also incorporates updated evidence from RCTs, offering a more comprehensive and current synthesis of the biological mechanisms of exercise. By analyzing a broader range of RCTs, we aim to contribute to a clearer understanding of the neurobiological effects of physical exercise in adult MDD patients.

## Materials and methods

This systematic review was conducted following the Preferred Reporting Items for Systematic Reviews and Meta-Analyses (PRISMA) guidelines (Page et al., 2021) and was registered on PROSPERO (CRD42024548511). See the literature search in Supplementary Materials.

### Eligibility criteria

All retrieved records were imported into Zotero for reference management, and duplicates were removed. Two independent reviewers screened titles and abstracts. Disagreements on inclusion were resolved by a third reviewer. Abstracts lacking sufficient inclusion or exclusion information were assessed in full text.

Studies were included if they met the following criteria: (1) RCTs evaluating physical exercise interventions and their effects on biomarkers of any biological system, with pre- and post-intervention blood markers. Only RCTs with a control group or those comparing two or more interventions were considered. Exercise was defined as a planned, structured, and repetitive intervention aimed at improving or maintaining physical conditioning; (2) conducted in adults aged 18 or older, diagnosed with MDD according to the Diagnostic and Statistical Manual of Mental Disorders (DSM)-IV, DSM-IV-TR, DSM-5, International Classification of Diseases (ICD)-10, and ICD-11 criteria; and (3) published in English.

Exclusion criteria were: (1) studies involving children and adolescents or those focusing on conditions other than MDD; (2) non-RCTs; (3) RCTs on physical exercise in MDD patients without pre- and post-intervention biomarker data.

### Data extraction

Data were extracted following PRISMA guidelines and included authors, year of publication, study design, sample size, participant characteristics (age, gender, socioeconomic status), diagnostic criteria, medications, treatment duration, inpatient/outpatient status, exercise intervention details (type, duration, intensity, supervision), depression and symptom measures, biomarkers (pre- and post-intervention), assay type, and manufacturer. The primary outcome was the identification of the biological effects of physical exercise in MDD patients. Secondary outcomes included changes in depression and related symptoms following exercise interventions.

### Risk of bias (quality) assessment

The Revised Cochrane Risk of Bias (RoB 2) tool for randomized trials was used to assess methodological quality and internal validity (Sterne et al., 2019). This tool evaluates bias across five domains: randomization process, deviations from intended interventions, missing outcome data, measurement of outcomes, and selection of reported results. The overall study quality was coded as low, of some concerns, or high. Two independent researchers did the study quality assessments, discussing with a third reviewer to resolve inconsistencies.

## Results

### Characteristics of the included studies

**Figure 1.**
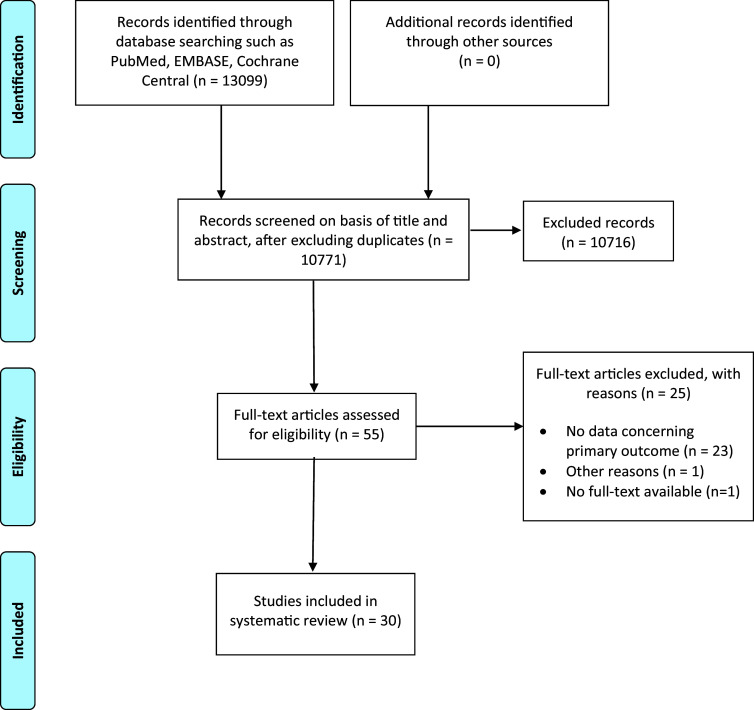
Flowchart showing the study selection procedure.

A flowchart of the search process is shown in Figure 1, whereas the main characteristics of the included studies are summarized in Table 1. A total of 2194 subjects were included. The mean age in the exercise intervention groups was 42.3 years, compared to 42.8 years in the control groups. The percentage of females in the intervention and control groups was 68.5% and 65%, respectively.

**Table 1.** Summary of the main characteristics of the included randomized controlled trials

Authors/year/country	Sample size	Gender (F %)	Mean age (years)	Type of exercise	Intensity of exercise	Days of exercise per week	Intervention duration (in weeks)	Biological parameters of interest
Carneiro et al. (2016), Portugal	Exercise: 7/control: 7	Exercise (100%)/control (100%)	Exercise: 55.0/control: 47.9	Aerobic	Moderate	3	16	COMT (monoaminergic system)
Carneiro et al. (2017), Portugal	Exercise: 9/control: 10	Exercise (100%)/control (100%)	Exercise: 52.8/control: 47.8	Aerobic	Moderate	3	16	Monoamines Stress-response markers
Euteneuer et al. (2017), Germany	CBT-Exercise: 34/CBT-control: 34/control: 30	CBT-exercise (44.5%)/CBT-control (54.3%)/Control (43.4%)	Exercise: 36.9/Control: 37.9	Aerobic	Moderate	4	16	Inflammatory and immune biomarkers
Fernandes, Siqueira, et al. (2022), Brazil	Exercise: 20/control: 20	AD + exercise (80%)/AD (90%)	Exercise: 43.5/control: 38.6	Aerobic	High	4	4	Inflammatory and immune biomarkers
Gerber et al. (2020), Switzerland	Exercise: 14/control: 11	Exercise (42.9%)/control (63.7%)	Exercise: 39.4/control: 36.4	Aerobic	Moderate	3	6	Stress-response markers
Hennings et al. (2013), Germany	Exercise: 38/control: 48	Exercise (60.5%)/control (66.7%)	Exercise: 32.1/control: 36.4	Aerobic	Moderate	5	4	Inflammatory and immune biomarkers Monoamines
Imboden et al. (2021), Switzerland	Exercise: 22/control: 20	Exercise (45.5%)/control (50.0%)	Exercise: 41.3/control: 38.3	Aerobic	Moderate	3	6	Inflammatory and immune biomarkers Stress-response markers
Kerling et al. (2017), Germany	Exercise: 22/control: 20	Exercise (45.5%)/control (30.0%)	Exercise: 44.2/control: 40.9	Aerobic	Moderate	3	6	BDNF
Krogh, Benros, et al. (2014), Denmark	Exercise: 56/control: 59	Exercise (71.4%)/control (62.7%)	Exercise: 39.7/control: 43.4	Aerobic	Moderate	3	12	Inflammatory and immune biomarkers
Krogh et al. (2013), Denmark	Exercise: 53/control: 58	Not available	Not available	Aerobic	Moderate	3	12	Copeptin
Krogh et al. (2010), Denmark	Exercise: 31/strength training: 29/relaxation program: 28	Exercise (83.9%)/strength (89.7%)/relaxation (42.9%)	Exercise: 39.3/strength: 42.4/relaxation: 36.0	Aerobic	Moderate/high	2	16	Endocrine markers stress-response markers
Krogh, Rostrup, et al. (2014), Denmark	Exercise: 41/control: 38	Exercise (73.2%)/control (60.5%)	Exercise: 38.9/control: 43.8	Aerobic	Moderate	3	12	Neurotrophic markers
Lavretsky et al. 2011), USA	Exercise: 33/control: 35	Tai Chi Chih (69.7%)/control (62.9%) (baseline data)	Exercise: 69.1/control: 72 (baseline data)	Non-aerobic	Low	2	10	Inflammatory and immune biomarkers
Nugent et al. (2021), USA	Exercise: 48/control: 39	Exercise (91.7%)/control (74.4%)	Exercise: 45.5/control: 44.8	Non-aerobic	Moderate/low	2	10	Inflammatory and immune biomarkers
C. D. Rethorst et al. (2015), USA	105 MDD patients randomized for 16 vs. 4 KKW exercise	80% (data refer to the whole sample)	47.5 (data refer to the whole sample)	Aerobic	Exercise 4 KKW; Exercise 16 KKW		12	Inflammatory and immune biomarkers BDNF
Chad D. Rethorst et al. (2017), USA	122 MDD patients randomized for 16 vs. 4 KKW for exercise	Not available	Not available	Aerobic	Exercise 4 KKW; Exercise 16 KKW		12	Inflammatory and immune biomarkers BDNF
C. D. Rethorst et al. (2013), USA	Exercise 16 KKW: 53/Exercise 4 KKW: 52	Exercise 16 KKW (80%)/Exercise 4 KKW (75%)	Exercise 16 KKW: 45.8/Exercise 4 KKW: 49.2	Aerobic	Exercise 4 KKW; Exercise 16 KKW	Changed each week	12	Inflammatory and immune biomarkers
Dabidy Roshan et al. (2011), Iran	Exercise: 12/control: 12	Exercise (100%)/control (100%)	Exercise: 16.9/control: 16.8	Aerobic	Moderate	3	6	Monoamines
Salehi et al. (2016), Iran	ECT: 20/ECT + exercise: 20/Exercise: 20	ECT (35%)/ECT + exercise (35%)/Exercise: (20%)	ECT: 29.7/ECT + exercise: 30.2/Exercise: 29.0	Aerobic	Moderate	3	4	BDNF
Sarubin et al. (2014), Germany	Exercise: 22/control: 31	Exercise (36.4%)/control (22.6%)	Exercise: 37.2/control: 42.3	Non-aerobic	Low	1	5	Stress-response biomarkers
Schuch et al. (2014), Brazil	Exercise: 15/control: 11	Exercise (73.4%)/control (72.7%)	Exercise: 42.8/control: 42.5	Aerobic	Moderate-high	3	3	Oxidative stress markers BDNF
Shi and Miao (2017), China	Exercise 20 KKW: 50/exercise 5 KKW: 45	Exercise 20 KKW (85%)/exercise 5 KKW (75%)	Exercise 20 KKW: 44.8/exercise 5 KKW: 49.3	Aerobic	Different intensities		15	Inflammatory and immune biomarkers
Siddarth et al. (2023), USA	Exercise: 55/control: 58	Exercise (69.4%/control (75.3% (data refer to baseline, total of 85 participants)	Exercise: 69/control: 69.5 (data refer to baseline, total of 85 participants)	Non-aerobic	Low	1	12	Inflammatory and immune biomarkers
Szuhany and Otto (2020), USA	Exercise: 14/control: 15	Exercise (64.2%)/Control (86.7%)	Exercise: 31/Control: 37.2	Aerobic	Moderate	Nine sessions over 12 weeks	12	BDNF
Tolahunase, Sagar, and Dada (2018), India	Yoga: 89/pharmacotherapy: 89	Yoga (47.2%)/pharmacotherapy (48.3%)	Yoga: 38/pharmacotherapy: 40	Non-aerobic	Low	5	12	Serotonin genetic polymorphisms
Tolahunase, Sagar, Faiq, et al. (2018), India	Yoga: 29/control: 29	Yoga (55.2%)/control (51.7%)	Yoga: 36.9/control: 39.1	Non aerobic	Low	5	12	Inflammatory and immune biomarkers Neurotrophic markers Oxidative stress markers
Torelly et al. (2022), Brazil	33 MDD patients randomized to: mind–body practice, control, exercise × exercise, control, mind–body practice	72.7% (data refer to the whole sample)	Mean age: 37 (data refer to the whole sample)	Aerobic and non-aerobic	Moderate/low		1	Inflammatory and immune biomarkers
Toups et al. (2011), USA	Exercise 16 KKW: 52/exercise 4 KKW: 52	Exercise 16 KKW (84%)/exercise 4 KKW (75%)	Exercise 16 KKW: 46.1/exercise 4 KKW: 49.2	Aerobic	Moderate	Exercise 16 KKW; exercise 4 KKW	12	BDNF
Wang and Li (2022), China	Exercise: 30/control: 30	Not available	Not available	Aerobic	Moderate	Three to five times a week	8	β-endorphin
Krogh et al. (2012), Denmark	Exercise: 56/control: 59	Exercise (71.4%)/control (62.7%)	Exercise: 39.7/control: 43.4	Aerobic	Moderate	3	12	Metabolic markers

Abbreviations: AD: antidepressant; BDNF: brain-derived neurotrophic factor; CBT: cognitive behavior therapy; COMT: catechol-O-methyltransferase; ECT: electroconvulsive therapy; F: female; KKW: kilocalories per kilogram of bodyweight per week; MDD: major depressive disorder.

Information on illness duration could not be systematically reported, as it was often missing or inconsistently described across the included studies. The clinical course and duration of depression varied widely, ranging from single moderate episodes to chronic, recurrent forms of depression. All included studies confirmed the diagnosis of depression using standardized diagnostic instruments.

### Main findings Inflammatory and immune systems

Fifteen studies exploring the effects of physical exercise on inflammatory and immune biomarkers in MDD were included.

Exercise combined with cognitive-behavioral therapy (CBT) significantly increased anti-inflammatory IL-10 levels in patients with MDD (Euteneuer et al., 2017). However, other studies found no changes in IL-10 levels in MDD patients submitted to exercise in comparison to controls (Fernandes, Siqueira, et al., 2022; Siddarth et al., 2023). Lower baseline levels of the pro-inflammatory IL-6 predicted treatment non-response in patients with MDD undergoing two exercise doses for 12 weeks (Chad D. Rethorst, South, Rush, Greer, & Trivedi, 2017). IL-6 levels significantly reduced after hatha yoga intervention in comparison with health education control in depressed patients (Nugent et al., 2021). In addition, yoga and meditation-based lifestyle interventions reduced IL-6 levels in comparison to controls in patients with MDD (Tolahunase, Sagar, Faiq, & Dada, 2018). In other findings, although elevated IL-6 levels were observed in patients with MDD, this marker was not significantly influenced by exercise intervention (Krogh et al., 2014). Also, other results did not show significant changes in IL-6 following exercise in addition to pharmacological treatment in depressed patients in comparison with drug therapy alone (Fernandes, Siqueira, et al., 2022; Siddarth et al., 2023) or following exercise intervention in patients with MDD versus controls (Hennings et al., 2013). Other cytokines were also studied. No significant changes in IL-12, IL-8, and IL-1β were observed following exercise intervention in comparison to controls in patients with MDD (Fernandes, Siqueira, et al., 2022). Similarly, no significant IL-1β changes were observed following a 15-week exercise intervention in MDD patients (Shi & Miao, 2017). A large panel of cytokines was analyzed by Siddarth and colleagues comparing patients with MDD undergoing Tai Chi Chih (TCC) or health education, with no differences between groups (Siddarth et al., 2023). However, exercise and mind–body practices reduced IL-1β levels in MDD patients (Torelly, Novak, Bristot, Schuch, & Pio de Almeida Fleck, 2022). Also, reductions in hypersomnia were linked to decreases in IL-1β following exercise in patients with MDD, suggesting a relationship between cytokine changes and depressive symptoms after exercise (C. D. Rethorst et al., 2015). These findings suggest that specific ILs, particularly IL-6, IL-10, and IL-1β, may be differentially affected by exercise and mind–body interventions, with varying results.

Exercise and mind–body practices showed mixed findings on CRP. Exercise combined with CBT marginally reduced CRP levels in depressed patients, particularly in those with cardiovascular risk (Euteneuer et al., 2017). Reduced CRP levels were observed in elderly MDD patients undergoing TCC alongside escitalopram, supporting the notion that physical and mind–body practices may reduce inflammation in subgroups of depressed individuals (Lavretsky et al., 2011). In contrast, no significant changes in CRP levels were found in depressed individuals following hatha yoga intervention (Nugent et al., 2021), and no differences in CRP levels were found between aerobic and stretching exercise programs (Krogh et al., 2012). Elevated CRP levels were observed in MDD patients, but this marker was not significantly influenced by exercise (Krogh, Benros, et al., 2014).

Tumor necrosis factor-alpha (TNF-α) responses were similarly inconsistent. Baseline TNF-α levels showed a correlation with treatment response, with higher baseline levels predicting greater reductions in depressive symptoms during exercise (C. D. Rethorst et al., 2013), suggesting that TNF-α may predict treatment efficacy. An acute decrease in TNF-α levels after a single session of mixed aerobic and anaerobic exercise was reported by Torelly et al. (2022). On the contrary, other authors found no significant changes in TNF-α levels following hatha yoga (Nugent et al., 2021) or TCC (Siddarth et al., 2023), and no differences were observed between aerobic exercise and controls for this biomarker (Imboden et al., 2021). These findings underline the complexity of TNF-α as a biomarker of exercise in MDD, with its responsiveness varying depending on the type and duration of the intervention.

### Neurotrophic system

Twelve studies investigated the effects of exercise on BDNF, a neurotrophic factor linked to mood regulation and neuroplasticity, in exercise interventions for MDD. Aerobic exercise for 6 weeks significantly increased serum BDNF concentrations in patients with MDD (Kerling et al., 2017). Mind–body practices and physical exercise increased BDNF levels in MDD patients, with a time-dependent effect (Torelly et al., 2022). Similarly, other results showed increased BDNF levels following exercise, although these changes did not directly correlate with improvements in depressive symptoms (Szuhany & Otto, 2020). Higher BDNF levels predicted remission, while lower levels of this marker predicted non-response in MDD patients randomized for two exercise doses over 12 weeks (Chad D. Rethorst et al., 2017). Conversely, BDNF reductions were associated with improved hypersomnia in non-remitted MDD patients (C. D. Rethorst et al., 2015). Other studies showed mixed results regarding the relationship between exercise and BDNF levels in MDD. An increase in serum BDNF levels was observed during a 6-week inpatient treatment program for depression, with no significant differences between the aerobic exercise and stretching groups (Imboden et al., 2021). Similarly, no significant changes were found in BDNF levels in MDD patients following a 3-month exercise intervention compared to controls, despite improved aerobic capacity (Krogh et al., 2014). Other findings showed no significant changes in BDNF levels with exercise, despite improvements in depressive symptoms, highlighting the variability in BDNF responses to physical activity (Schuch et al., 2014).

Baseline BDNF moderated response to exercise in MDD patients partially responding to antidepressants, with higher baseline levels linked to faster symptom improvement, even though overall BDNF levels did not correlate with depression improvement (Toups et al., 2011). Significant BDNF increases and symptom improvements were observed with electroconvulsive therapy (ECT), aerobic exercise, and combined treatment in MDD patients, with the highest remission rates found in the ECT combined with exercise (Salehi et al., 2016). Significant correlations between depressive symptoms decrease and increased BDNF levels were found in MDD patients undergoing yoga- and meditation-based lifestyle intervention compared to the controls (Tolahunase, Sagar, Faiq, et al., 2018).

Studies on other neurotrophic factors, such as vascular endothelial growth factor (VEGF) and insulin-like growth factor 1 (IGF-1), provided inconsistent results. No significant effects of aerobic exercise were found on VEGF or IGF-1 levels in MDD patients, despite memory and depressive symptoms improvements (Krogh, Rostrup, et al., 2014). No significant VEGF changes were found in depressed adults undergoing TCC versus controls (Siddarth et al., 2023). These findings suggest that VEGF and IGF-1 may not be primary targets for exercise interventions in MDD, although they could play a role in cognitive improvements associated with exercise. Other factors, including epidermal growth factor (EGF), fibroblast growth factor-2, and transforming growth factor-α, were also unaffected by TCC in MDD patients (Siddarth et al., 2023).

### Stress-response system

Eight studies on the effects of exercise on the stress-response system in MDD were included. Cortisol, a key stress-response marker, is widely studied in this context. Six weeks of aerobic exercise did not significantly alter cortisol reactivity to a laboratory stress task in MDD patients, many of whom displayed blunted baseline cortisol responses, indicating a limited impact of exercise on hypothalamic–pituitary–adrenal (HPA) axis reactivity (Gerber et al., 2020). Similarly, overall cortisol reductions during antidepressant treatment showed no significant differences between patients engaging in physical exercise and those who did not, revealing limited additional effects of exercise on cortisol regulation in MDD (Fernandes, Siqueira, et al., 2022). Adding yoga to quetiapine or escitalopram treatment did not significantly enhance HPA axis downregulation beyond medication alone (Sarubin et al., 2014), and exercise combined with pharmacotherapy did not alter cortisol levels compared to pharmacotherapy alone (Carneiro et al., 2017). Krogh and colleagues found no effects of strength or aerobic training on resting cortisol levels or cortisol responses to acute exercise stress in MDD patients versus controls (Krogh, Nordentoft, Mohammad-Nezhad, & Westrin, 2010). However, yoga and meditation significantly reduced cortisol levels in the intervention group in comparison with controls (Tolahunase, Sagar, Faiq, et al., 2018).

Research on the cortisol awakening response (CAR) presented mixed results. A decreased CAR over time was observed in moderate-to-severe MDD patients during inpatient treatment, regardless of whether aerobic exercise or stretching was used, indicating that the decline may be treatment-related rather than exercise-specific (Imboden et al., 2021). In contrast, Krogh et al. found no significant associations between cortisol, adrenocorticotropic hormone, or copeptin in depressed patients at rest or after acute exercise, indicating that these stress markers do not consistently respond to exercise interventions. Additionally, copeptin levels showed no significant differences between depressed patients and healthy controls, further indicating it may not play a critical role in exercise responses (Krogh et al., 2013).

### Monoaminergic system

The relationship between physical exercise, MDD, and monoaminergic biomarkers has also been studied, although findings remain inconsistent. Our review included four studies, with one genetic study and others focusing on candidate protein biomarkers in peripheral blood.

A genetic study explored serotonin-related gene polymorphisms, specifically the serotonin transporter-linked polymorphic region (5-HTTLPR) within the serotonin transporter gene (*SLC6A4*) and MTHFR 677C > T, in MDD patients. Yoga significantly increased remission odds compared to pharmacotherapy, regardless of genotype. Interestingly, childhood adversity interacted with these polymorphisms to reduce treatment response in the pharmacotherapy group but not in the yoga arm (Tolahunase, Sagar, & Dada, 2018).

Carneiro and colleagues found that combining exercise with pharmacotherapy reduced the activity of soluble catechol-O-methyltransferase, and enzyme involved in catecholamine metabolism and relevant to mood regulation (Carneiro et al., 2016). However, other authors reported no significant changes in plasma levels of monoamine neurotransmitters, such as serotonin, dopamine, noradrenaline, or adrenaline, when aerobic exercise was added to pharmacotherapy in depressed women (Carneiro et al., 2017), highlighting the complexity of exercise’s effects on monoamines in MDD.

Regarding serotonin metabolism, short-term graded exercise did not alter serotonergic markers such as tryptophan, kynurenine, or 5-hydroxyindoleacetic acid in patients with MDD or somatoform syndrome (Hennings et al., 2013). Exercise did not significantly affect serotonergic parameters, especially in short durations or when used as an isolated intervention.

For noradrenaline, Dabidy Roshan and colleagues found increased 24-hour urinary MHPG sulfate, a noradrenaline metabolite, after intermittent water-walking exercise, correlating with symptom reduction (Dabidy Roshan, Pourasghar, & Mohammadian, 2011). These findings suggest the noradrenergic system may mediate mood improvements linked to exercise, although the mechanisms remain unclear.

### Other systems

Other biological systems, including the endocrine system (two studies), oxidative stress markers (two studies), and β-endorphins (one study), have also been examined.

The impact of exercise on the endocrine system in MDD shows mixed results. Altered growth hormone (GH) and cortisol responses to acute exercise were observed in MDD patients compared to controls, but no significant changes in resting hormone levels occurred over time (Krogh et al., 2010). Conversely, aerobic exercise improved metabolic markers, including lower fasting glucose levels and reduced waist circumference in MDD patients, although without significant antidepressant effects (Krogh et al., 2012).

Exercise’s ability to reduce oxidative stress was supported by findings of reduced thiobarbituric acid-reactive substances, a marker of oxidative stress, in severely depressed inpatients following exercise (Schuch et al., 2014), supporting that exercise can reduce oxidative damage. Yoga- and meditation-based interventions also decreased oxidative stress markers in MDD patients, highlighting the benefits of these practices (Tolahunase, Sagar, Faiq, et al., 2018).

Regarding β-endorphins, exercise combined with antidepressant therapy increased serum β-endorphin levels, correlating with greater reductions in depression severity in the intervention versus control groups (Wang & Li, 2022). This suggests that exercise-induced β-endorphin release may enhance clinical outcomes.

The main findings of the included studies are summarized in Table 2, which provides an overview of the effects of physical exercise on various biological systems in patients with MDD.

**Table 2.** Summary of the main findings

Inflammatory/immune system	Neurobiological findings	Studies
IL–10 (anti-inflammatory)	MDD patients in exercise showed higher plasma IL–10 levels (*t*_69_ = −2.96; *p* = 0.004) than controls.	Euteneuer et al. (2017)
	No significant changes in plasma IL–10 levels in exercise vs. control groups	Fernandes, Siqueira, et al. (2022) Siddarth et al. (2023)
IL–6 (pro-inflammatory)	In MDD patients randomized to 16 KKW vs. 4 KKW exercise, lower serum IL–6 levels predicted non-response.	Chad D. Rethorst et al. (2017)
	Decreased plasma IL–6 levels were observed in Yoga group vs. controls in MDD patients (β_s-IL–6_ = −0.24, SE = 0.09, *p* < 0.05).	Nugent et al. (2021)
	Decreased IL–6 levels were observed in Yoga group vs. controls in depressed patients (*p* < 0.001).	Tolahunase, Sagar, Faiq, et al. (2018)
	No significant changes in plasma IL–6 levels in exercise vs. controls in MDD patients	Krogh, Benros, et al., 2014 Fernandes, Siqueira, et al. (2022) Siddarth et al. (2023)
IL–1β (pro-inflammatory)	In MDD patients, serum IL–1β increased over time with exercise (*p* = 0.015); positive affect correlated with IL–1β reduction in mind–body (*r* = −0.54, *p* < 0.05) and exercise groups (*r* = −0.48, *p* < 0.05).	Torelly et al. (2022)
	Hypersomnia reduction was associated with lower IL–1β post-exercise in MDD (*ρ* = 0.37, *p* = 0.002).	C. D. Rethorst et al. (2015)
	No significant changes in plasma IL–1β levels in exercise × controls in MDD patients.	Fernandes, Siqueira, et al. (2022)
	In MDD patients, IL–1β changes correlated with depression scores (rs = 0.24, *p* < 0.05), especially in high exercise dose (rs = 0.38, *p* < 0.05) vs. low dose.	C. D. Rethorst et al. (2013)
	In MDD patients, IL–1β changes correlated with symptom scores (rs = 0.24, *p* = 0.044), notably in the high exercise dose (rs = 0.32, *p* < 0.05).	Shi and Miao (2017)
IL–12 (pro-inflammatory)	No significant changes in plasma IL–12 levels in exercise vs. controls in MDD patients	Fernandes, Siqueira, et al. (2022
IL–8 (pro/anti-inflammatory)	No significant changes in plasma IL–8 levels in exercise vs. controls in MDD patients	Fernandes, Siqueira, et al. (2022)
Other cytokines	No significant changes in plasma levels of cytokines (IL–3, IL–4, IL–5, IL–7, IL–9, IL–12, IL–13, IL–15, IL–17, IL–19) in TCC vs. controls in MDD patients	Siddarth et al. (2023)
CRP	CBT-exercise was associated with lower CRP levels (*t*_80_ = 2.75; *p* = 0.007) vs. CBT without exercise.	Euteneuer et al. (2017)
	Lowers plasma CRP in TCC vs. health education in MDD patients (time effect: F_[2, 78]_ = 3.14, *p* < 0.05)	Lavretsky et al. (2011)
	No significant changes in plasma CRP levels in Yoga vs. health education in MDD patients	Nugent et al. (2021)
	No significant changes in plasma CRP for exercise vs. control in MDD patients	Krogh et al. (2012) Krogh, Benros, et al. (2014)
TNF-α	In MDD patients, higher baseline TNF-α predicted greater depression score reduction (parameter estimate = −0.6155, *p* = 0.002), with no significant post-exercise TNF-α changes or dose-related effects.	C. D. Rethorst et al. (2013)
	Exercise reduced serum TNF-α levels in exercise vs. control in MDD patients (*p* = 0.003).	Torelly et al. (2022)
	No significant changes in plasma TNF-α for yoga vs. controls in MDD (β_s-TNF-α_ = −0.01, SE = 0.08, *p* > 0.05)	Nugent et al. (2021)
	No significant changes in plasma TNF-α for TCC vs. health education in MDD patients	Siddarth et al., 2023
	No significant changes in serum TNF-α for exercise vs. control in MDD patients (Eta^2^ = 0.001, *p* = 0.977)	Imboden et al. (2021)
**Neurotrophic system**		
BDNF	In MDD patients, serum BDNF decreased in TAU and increased in exercise (F = 5.05, *p* = 0.030).	Kerling et al. (2017)
	In MDD patients, serum BDNF levels increased in exercise vs. controls (*p* = 0.003).	Torelly et al. (2022)
	In MDD patients, serum BDNF increased in exercise vs. stretching groups (d = 0.83, *p* < 0.001).	Szuhany and Otto (2020)
	In MDD patients, exercise showed no additional effects on serum BDNF; however, BDNF improved during inpatient treatment (Eta^2^ = 0.201, *p* = 0.014).	Imboden et al. (2021)
	Significant increase in BDNF levels in Yoga vs. controls in depressed patients (*p* < 0.001)	Tolahunase, Sagar, Faiq, et al. (2018)
	In MDD patients randomized for 16 KKW or 4 KKW exercise, higher serum BDNF levels predicted remission and lower serum BDNF levels predicted treatment nonresponse.	Chad D. Rethorst et al. (2017)
	In MDD patients randomized for 16 KKW or 4 KKW exercise, reduction in hypersomnia was correlated with reductions in serum BDNF (Spearman *ρ* = 0.26, *p* = 0.029).	C. D. Rethorst et al. (2015)
	No significant changes in BDNF serum levels for exercise vs. control condition in MDD (*p* = 0.47)	Krogh, Rostrup, et al. (2014)
	In depressed patients randomized to exercise × TAU, significant time effect for BDNF (corrected *p* < 0.001) was found, but no group × time interaction (*p* = 0.13) was observed with added exercise.	Schuch et al. (2014)
	In MDD patients randomized for 16 KKW or 4 KKW exercise, resting serum BDNF did not change (*p* = 0.15), higher baseline BDNF predicted faster symptom improvement (*p* = 0.003), and baseline BDNF levels moderated exercise effect through BMI interaction (BDNF by BMI by time effect: F = 7.15, *p* = 0.009).	Toups et al. (2011)
	In MDD patients randomized to ECT, ECT + exercise or exercise, increased plasma BDNF levels were observed (*p* < 0.001), with higher effects in the ECT + exercise group.	Salehi et al. (2016)
Other neurotrophic factors	No significant changes in VEGF (*p* = 0.86) and IGF–1 (*p* = 0.54) serum levels after exercise vs. controls in MDD patients	Krogh, Rostrup, et al. (2014)
	No significant VEGF, EGF, FGF–2 and TGF-α plasma levels changes post-TCC vs. controls in MDD. Plasma EGF increased in both groups in remitters vs. non-remitters (F_(1,111)_ = 4.74, effect size = 0.49, *p* = 0.03).	Siddarth et al. (2023)
**Stress-response system**		
	No significant changes in salivary cortisol after stress test in exercise vs. controls in MDD (*p* = 0.860)	Gerber et al. (2020)
	In MDD patients randomized for exercise + pharmacotherapy vs. pharmacotherapy only, plasma cortisol decreased with pharmacotherapy (η^2^ = 0.112, *p* = 0.035), without differences between groups.	Fernandes, Siqueira, et al. (2022)
	In MDD patients, yoga vs. control showed no long-term cortisol difference; both groups had decreased cortisol in DEX/CRH test.	Sarubin et al. (2014)
	No significant changes on cortisol plasma levels after exercise vs. controls in MDD patients	Carneiro et al. (2017)
	Plasma cortisol response to exercise differed in MDD patients vs. controls (F_4, 149_ = 2.399, *p* = 0.05).	Krogh et al. (2010)
	Decreased cortisol levels were observed in Yoga vs. controls in MDD patients (*p* < 0.001).	Tolahunase, Sagar, Faiq, et al. (2018)
	In MDD patients randomized for exercise vs. controls, salivary cortisol during CAR increased over time, with time effect on AUC_total_ (Eta^2^ = 0.307, *p* = 0.001) and AUC_basal_ (Eta^2^ = 0.201, *p* = 0.014) across groups.	Imboden et al. (2021)
	No significant difference in copeptin levels at rest or after exercise in MDD patients vs. controls; copeptin decreased at rest with high aerobic training compliance (*p* = 0.04).	Krogh et al. (2013)
**Monoaminergic system**		
	In depressed patients, Yoga showed significant odds of remission vs. pharmacotherapy; depression symptom reduction was lower in SS vs. LL genotypes for 5-HTTLPR (*p* = 0.025) in drug group. Childhood adversity decreased treatment response in drug arm (*p* = 0.02), and not in Yoga (*p* = 0.78).	Tolahunase, Sagar, and Dada (2018)
	Exercise + pharmacotherapy reduced soluble COMT activity in MDD vs. controls (r = 0.535, *p* = 0.02).	Carneiro et al. (2016)
	No significant changes in plasma serotonin, dopamine, noradrenaline, or adrenaline after aerobic exercise added to pharmacotherapy in depressed women vs. controls	Carneiro et al. (2017)
	No associations between symptom improvement and serotonergic markers in MDD, somatoform, and controls after exercise; significant group effect for kynurenine (F = 3.81, η^2^ = 0.07, *p* < 0.05)	Hennings et al. (2013)
	In depressed patients, urine MHPG sulfate increased with exercise vs. controls (*p* ≤ 0.001) and negatively correlated with depression symptoms (r = −0.65, *p* ≤ 0.001).	Dabidy Roshan et al. (2011)
**Other systems**		
	Plasma GH response differed in depressed patients vs. controls (F_4,168_ = 2.916, *p* = 0.02); GH decreased in strength-training (*p* = 0.03) vs. aerobic and relaxation; prolactin response to exercise was similar in depressed patients vs. controls (F_4,155_ = 0.74, *p* = 0.56).	Krogh et al. (2010)
	Exercise group had lower blood glucose levels vs. stretching control in depressed patients (mean difference: 0.2 (0.0–0.5), *p* = 0.04).	Krogh et al. (2012)
	In depressed patients randomized to exercise vs. TAU, significant group × time interaction was observed for TBARS serum levels (*p* = 0.02). Exercise decreased TBARS in MDD (corrected *p* = 0.01).	Schuch et al. (2014)
	Yoga decreased DNA damage and balanced oxidative stress markers vs. controls in MDD (*p* < 0.001).	Tolahunase, Sagar, Faiq, et al. (2018)
	In depressed patients, exercise augmentation showed greater symptom improvement and higher serum β-endorphin levels vs. TAU.	Wang and Li (2022)

Abbreviations: 5-HTTLPR: serotonin-transporter-linked promoter region; ACTH: adrenocorticotropic hormone; AUC: area under the curve; BDNF: brain derived neurotrophic factor; BMI: body mass index; CAR: cortisol awakening response; CBT: cognitive behavior therapy; COMT: catechol-O-methyltransferase; CRP: C-reactive protein; DEX/CRH: dexamethasone/corticotropin releasing hormone; ECT: electroconvulsive therapy; EGF: epidermal growth factor; FGF: fibroblast growth factor; GH: growth hormone; HPA: hypothalamic pituitary adrenal axis; IGF: insulin-like growth factor; IL: interleukin; KKW: kilocalories per kilogram of bodyweight per week; MDD: major depressive disorder; MTHFR: methylenetetrahydrofolate reductase; SE: standard error; TAU: treatment as usual; TCC: Tai Chi Chih; TBARS: thiobarbituric acid-reactive substances; TNF-α: tumor necrosis factor-alpha; VEGF: vascular endothelial growth factor.

### Biomarkers as potential predictors of treatment response

Higher baseline levels of TNF-α were associated with greater reductions in depressive symptoms during exercise treatment in patients with MDD (C. D. Rethorst et al., 2013). Similarly, baseline levels of IL-6 were predictive of treatment response, with lower IL-6 levels identifying non-responders to a 12-week exercise program (Chad D. Rethorst et al., 2017). In the neurotrophic domain, higher baseline concentrations of BDNF were associated with a faster symptom improvement in patients partially responding to antidepressants who received adjunctive exercise (Toups et al., 2011). Additionally, BDNF levels at baseline predicted remission following a structured aerobic exercise program, with higher levels correlating with positive treatment outcomes (Chad D. Rethorst et al., 2017). Genetic variations also showed relevance in treatment stratification. Polymorphisms in 5-HTTLPR and MTHFR 677C > T interacted with a history of childhood adversity to predict reduced responsiveness to pharmacological interventions but were not associated with treatment outcomes in the yoga intervention group (Tolahunase, Sagar, & Dada, 2018).

### Quality of studies (RoB analyses)

The RoB evaluation for the included studies showed that 29 studies had an intermediate RoB, whereas one study had a high risk. In the analysis of each of the five domains, four studies had low risk, and 26 studies had some concerns in bias arising from the randomization process. For bias due to deviations from intended interventions, one study had a low risk, and 29 studies had some concerns. Bias due to missing outcome data was low in 29 studies, with one study showing some concerns. In bias related to outcome measurement, 26 studies had low risk, and four studies had some concerns. Finally, for bias in the selection of reported results, 20 studies had low risk, and 10 studies had some concerns. The main findings are summarized in Supplementary Figure 1.

## Discussion

In our systematic review, we contribute to the literature on the biological effects of physical exercise in MDD patients, highlighting various biological systems likely involved in exercise’s effects on MDD. The included studies showed mixed results.

For the inflammatory and immune systems, exercise combined with CBT increased IL-10 levels (Euteneuer et al., 2017), highlighting the potential of exercise to modulate immune responses, although other studies found no significant changes (Fernandes, Siqueira, et al., 2022; Siddarth et al., 2023). IL-6 reductions were associated with certain interventions (Nugent et al., 2021; Chad D. Rethorst et al., 2017; Tolahunase, Sagar, Faiq, et al., 2018), with other studies showing variable findings (Fernandes, Siqueira, et al., 2022; Krogh, Benros, et al., 2014; Siddarth et al., 2023). IL-1β responses were variable (C. D. Rethorst et al., 2015; Shi & Miao, 2017; Siddarth et al., 2023; Torelly et al., 2022), suggesting that cytokine modulation depends on the type of intervention and patient characteristics. In general, exercise and mind–body practices reduced IL-1β levels in MDD patients, pointing to an anti-inflammatory effect of these interventions. CRP exhibited limited responsiveness to exercise (Krogh, Benros, et al., 2014; Lavretsky et al., 2011; Nugent et al., 2021), with reductions in cardiovascular risk subgroups, suggesting a modest anti-inflammatory effect of the intervention (Euteneuer et al., 2017). These findings indicate that while CRP might be modulated by certain exercise interventions, its sensitivity as a biomarker in MDD treatment may be influenced by patient characteristics such as age or cardiovascular risk. TNF-α levels predicted treatment response (Rethorst et al., 2013), although the findings were inconsistent (Imboden et al., 2021; Nugent et al., 2021; Siddarth et al., 2023). Overall, findings related to the inflammatory and immune systems demonstrate that exercise and mind–body practices can influence inflammatory and immune biomarkers in patients with MDD. While ILs, CRP, and TNF-α are modulated by these interventions, their effects are not consistent across studies, highlighting the need for further research.

In the neurotrophic system, exercise increased BDNF levels, supporting physical activity as an effective adjunct therapy (Kerling et al., 2017; Salehi et al., 2016; Szuhany & Otto, 2020; Tolahunase, Sagar, Faiq, et al., 2018; Torelly et al., 2022), although its link to symptom improvement is unclear (Imboden et al., 2021; Krogh, Rostrup, et al., 2014; Schuch et al., 2014). The findings suggest that while exercise influences BDNF, its role in mediating clinical benefits may be complex. Other neurotrophic markers, including VEGF and IGF-1, were less consistently affected by exercise (Krogh, Rostrup, et al., 2014; Siddarth et al., 2023). In conclusion, BDNF is a key neurotrophic factor in depression treatment, but its role in mediating clinical improvements through exercise remains unclear. VEGF and IGF-1 may play less direct roles in MDD recovery, requiring further exploration. The neurotrophic system’s influence on MDD appears to be complex, depending on baseline characteristics and intervention types.

Within the stress-response system, cortisol showed limited sensitivity to exercise interventions (Carneiro et al., 2017; Fernandes, Siqueira, et al., 2022; Gerber et al., 2020; Krogh et al., 2010); however, mind–body practices, such as yoga, reduced cortisol levels (Tolahunase, Sagar, Faiq, et al., 2018). The effects of physical exercise on the stress-response system in MDD appear to be potential but limited, as most studies reported no significant changes in cortisol levels or reactivity, with only mind–body practices like yoga and meditation showing some promise in reducing cortisol, suggesting modest benefits of exercise on HPA axis regulation.

The monoaminergic system findings indicated that gene polymorphisms, particularly 5-HTTLPR and MTHFR 677C > T, interacted with childhood adversity to modulate treatment response (Tolahunase, Sagar, & Dada, 2018). Exercise also influenced catecholamine metabolism (Carneiro et al., 2016), but effects on serotonin and other monoamines were inconsistent (Carneiro et al., 2017; Hennings et al., 2013). The effects of physical exercise on monoaminergic biomarkers in MDD appear to be promising but limited, with inconsistent findings across studies. While some evidence suggests modest benefits on catecholamine metabolism and noradrenergic activity, other studies report no significant changes in serotonin-related markers, highlighting the need for further research to clarify these complex and variable responses.

In other systems, exercise influenced metabolic markers (Krogh et al., 2012); however, it did not consistently affect hormones like GH (Krogh et al., 2010). The results indicate that, although not translating into a significant antidepressant effect, exercise can have beneficial effects on metabolic aspects of the endocrine system. Exercise, yoga, and meditation-based interventions also reduced oxidative stress (Schuch et al., 2014; Tolahunase, Sagar, Faiq, et al., 2018), indicating the possible benefits of these practices. In summary, while endocrine responses to exercise remain inconsistent, metabolic improvements, such as reductions in oxidative stress and potential increases in β-endorphin levels (Wang & Li, 2022), are more consistently observed and may support better treatment outcomes. Further research is needed to clarify the conditions under which exercise most effectively modulates these biological markers.

Although there are systematic reviews and meta-analyses in this domain (da Cunha et al., 2023; Fernandes, Scotti-Muzzi, & Soeiro-de-Souza, 2022), our review is distinct in several aspects. While prior reviews focused on established biological systems involved in MDD and physical exercise, such as the inflammatory/immune and neurotrophic systems, our study employed a broader approach by considering additional biological systems. This strategy allows us to examine the biological effects of physical exercise from multiple perspectives, uncovering interactions and mechanisms not sufficiently reviewed in the literature. Indeed, our review expands on previous analyses by examining less-explored biological systems beyond immune-inflammatory and neurotrophic pathways, including oxidative stress, monoaminergic systems, and stress-response mechanisms. An additional strength of our review is the updated search, including studies published after 2020, providing a more current perspective on this evolving field. It further integrates updated evidence from RCTs, providing a more comprehensive and contemporary understanding of the biological mechanisms underlying exercise in MDD.

Analyzing the included studies based on the RoB assessment revealed issues that need to be addressed. The RoB evaluation showed that most studies had an intermediate risk, with one classified as high risk. Concerns were prevalent in domains associated with the randomization process, deviations from intended interventions, and the selection of reported results, where many studies exhibited a moderate RoB. These findings emphasize the need for methodological improvements in these areas to enhance the reliability of randomized trial outcomes.

Regarding the characteristics of the included studies, all used a candidate gene approach, evaluating a few biomarkers, mainly biochemical ones. While biochemical markers are relevant, the exclusive focus on them is a limitation. Internal and external factors can influence biochemical markers, and focusing solely on these markers may simplify the biological complexity of exercise effects on MDD. Expanding the approach to include genetic, epigenetic, and gene expression studies provide a more comprehensive understanding of the biological impacts of physical exercise on MDD.

Additionally, most included studies concentrated on biomarkers related to the inflammatory, immune, and neurotrophic systems. Although this focus aligns with the known pathophysiology of MDD and the mechanisms through which exercise exerts its benefits, it may limit our understanding of the full spectrum of biological changes that may occur with physical exercise in MDD. Future research should consider a wider array of biomarkers and employ broader approaches, such as omics technologies, to evaluate other systems and biomarkers.

The small sample sizes in many included studies represent another limitation. Small samples reduce the statistical power to detect effects and, combined with the heterogeneity in study designs, complicate the interpretation and generalizability of findings. For example, the control groups varied greatly, with some studies using non-MDD participants or participants engaging in different intensities of activity. This variability in controls influences comparisons between groups and weakens the interpretability of the findings. Specifically, the methodological variability across the included studies likely contributed to the inconsistencies observed in the results. Differences in exercise protocols (e.g., type, intensity, duration), sample characteristics (e.g., age, sex, comorbidities), and timepoints and methods of biomarker collection may have introduced heterogeneity in outcomes. Additionally, many studies lacked standardized outcome measures and often treated biological results as secondary rather than primary outcomes, limiting the rigor of biomarker assessments. Variability in the control group design and insufficient blinding or allocation concealment in several trials, as revealed by the RoB assessment, limit direct comparisons across studies. These methodological differences underscore the need for more standardized protocols and higher-quality RCTs to improve the reproducibility and interpretation of biological findings.

Furthermore, many studies do not effectively report effect sizes, making it challenging to evaluate the real impact of significant findings. Without clear information on effect sizes, it becomes difficult to assess the clinical relevance of the biological changes, limiting the understanding of the practical implications of the results. This gap limits a comprehensive understanding of the strength and consistency of exercise’s effects on biomarkers in MDD, essential for clinical translation.

There is also variability in the characteristics of study populations among the included studies. Differences in age, ethnicity, comorbidities, and other confounding variables add limitations to the interpretation of results. Moreover, the outcomes evaluated across studies are heterogeneous, and often, the biological effects of exercise were secondary outcomes rather than primary outcomes.

In addition to the limitations inherent to the included studies, such as small sample sizes, methodological variability in intervention and control groups, exercise protocols, biomarker assessment methods, and inconsistent reporting of statistical results and effect sizes, our review has certain limitations. One concern is publication and results selection bias, as studies with significant or favorable findings are more likely to be published, overrepresenting positive outcomes. Additionally, the absence of a meta-analysis limits the quantitative synthesis of findings, restricting a comprehensive evaluation of overall effect sizes and their relevance to clinical practice.

In conclusion, our systematic review highlights the complexity of the biological effects of physical exercise in MDD patients, revealing significant heterogeneity across biological systems. While exercise influences biomarkers in the inflammatory, neurotrophic, stress-response, and monoaminergic systems, the results are inconsistent and depend on the intervention type, patient characteristics, and the biomarkers studied. By exploring a broader range of biological systems, our review identifies potential new mechanisms underlying the therapeutic effects of exercise. However, limitations such as small sample sizes, varied study designs, inconsistent reporting of results, and a predominant focus on biochemical markers reduce the generalizability of the findings. Future research should prioritize larger, more homogeneous RCTs with standardized methods, a broader range of biomarkers, and advanced omics approaches, such as genomics, proteomics, and metabolomics, to clarify the biological mechanisms of exercise. Addressing these considerations would improve the understanding of the pathways involved in the beneficial effects of exercise on MDD. Overall, the findings highlight the complexity of physical exercise’s influence on biomarkers in MDD, warranting further research to clarify its therapeutic potential.

## Supporting information

Carvalho Silva et al. supplementary material Carvalho Silva et al. supplementary material

## Data Availability

Data from the current study can be made available upon request to the corresponding author.

## Supplementary material

The supplementary material for this article can be found at http://doi.org/10.1017/S0033291725100743.
