# Supplementary material for: Disentangling the biological mechanisms underlying the effects of physical exercise in major depressive disorder: a comprehensive systematic review of randomized controlled trials
Source: Psychol Med. 2025 Jul 16;55:e197. doi: 10.1017/S0033291725100743 (PMC12315648; doi:10.1017/S0033291725100743)
Supplement: Carvalho Silva et al. supplementary material [file S0033291725100743sup001.zip › Supplementary Material.docx]

**Supplementary Materials**

**Methods – Detailed section**

*Literature search*

Searches were performed in PubMed, Embase, and the Cochrane Central Register of Controlled Trials (CENTRAL), along with manual searches of reference lists from included studies and relevant review articles. RCTs published in English were included without time restrictions. The “English language” filter was applied.

*Search strings used for each database*

The search was conducted on May 6, 2024 and retrieved 2357 articles from PubMed, 7081 articles from EMBASE, and 3661 articles from Cochrane Central Register of Controlled Trials (CENTRAL). The following strings were used to perform the systematic search in each database:

**Pubmed:**

("Depression"[Mesh] OR "Depressive Disorder, Major"[Mesh] OR "Antidepressive Agents"[Mesh] OR depress*[title] OR "depressive dis*"[tiab] OR "depressive symptom*"[tiab] OR antidepress*[tiab] OR MDD[tiab]) AND ("Exercise"[Mesh] OR "Exercise Therapy"[Mesh] OR "Physical Fitness"[Mesh] OR "Sports"[Mesh] OR exercise*[tiab] OR "physical activit*"[tiab] OR aerobic*[tiab] OR "physical train*"[tiab] OR fitness[tiab] OR sport*[tiab]) AND ("Biomarkers"[Mesh] OR biomarker*[tiab] OR marker*[tiab] OR blood[subheading] OR serum[tiab] OR "Molecular Probes"[Mesh] OR probe[tiab] OR probes[tiab] OR genetics[subheading] OR "Phenotype"[Mesh] OR phenotyp*[tiab] OR endophenotyp*[tiab] OR genet*[tiab] OR "Gene Expression"[Mesh] OR "gene expression*"[tiab] OR transcript*[tiab] OR "Methylation"[Mesh] OR methyl*[tiab] OR "Epigenomics"[Mesh] OR "Epigenesis, Genetic"[Mesh] OR epigen*[tiab] OR "RNA"[Mesh] OR RNA[tiab] OR "Proteome"[Mesh] OR "Proteomics"[Mesh] OR proteom*[tiab] OR "Metabolism"[Mesh] OR metabolism[subheading] OR metabol*[tiab] OR biological[tiab] OR neurobiological[tiab] OR "molecular mechanism*"[tiab] OR "Cytokines"[Mesh] OR cytokin*[tiab] OR interleuk*[tiab] OR inflammat*[tiab] OR "oxidative stress"[tiab] OR "Brain-Derived Neurotrophic Factor"[Mesh] OR BDNF[tiab] OR "brain derived neurotrophic factor"[tiab] OR neurotrophin*[tiab] OR "Kynurenine"[Mesh] OR kynurenine[tiab] OR "Tryptophan"[Mesh] OR tryptophan[tiab] OR "Reactive Oxygen Species"[Mesh] OR "reactive oxygen species"[tiab] OR "sex hormone*"[tiab] OR "Testosterone"[Mesh] OR testosterone[tiab] OR "Estrogens"[Mesh] OR estrogen*[tiab] OR "Hydrocortisone"[Mesh] OR cortisol[tiab] OR "Biogenic Monoamines"[Mesh] OR monoamine*[tiab] OR serotonin[tiab] OR dopamine[tiab] OR "Melatonin"[Mesh] OR melatonin[tiab] OR noradrenaline[tiab] OR "Endocannabinoids"[Mesh] OR endocannabinoid*[tiab] OR endo-cannabinoid*[tiab] OR endocanabinoid*[tiab] OR "anandamide"[Supplementary Concept] OR anandamide[tiab] OR "glyceryl 2-arachidonate"[Supplementary Concept] OR 2-arachidonoylglycerol[tiab] OR 2-arachidonoyl-glycerol[tiab] OR 2-arachidonyl-glycerol[tiab] OR 2-arachidonylglycerol[tiab]) AND ("Clinical Trial"[Publication Type] OR trial[tiab] OR trials[tiab] OR random*[tiab] OR "Random Allocation"[Mesh] OR "Control Groups"[Mesh] OR group[tiab] OR groups[tiab] OR controls[tiab] OR "Double-Blind Method"[Mesh] OR "Single-Blind Method"[Mesh] OR blind*[tiab] OR placebo[tiab] OR RCT[tiab] OR RCTs[tiab] OR CCT[tiab] OR CCTs[tiab] OR NCT0*[tiab] OR NCT1*[tiab] OR NCT2*[tiab] OR NCT3*[tiab] OR NCT4*[tiab] OR NCT5*[tiab] OR NCT6*[tiab] OR NCT7*[tiab] OR NCT8*[tiab] OR NCT9*[tiab])

2357 results on May 6, 2024

**Embase**

('depression'/exp OR 'antidepressant agent'/exp OR 'depress*':ti OR 'depressive dis*':ti,ab,kw OR 'depressive symptom*':ti,ab,kw OR 'antidepress*':ti,ab,kw OR 'MDD':ti,ab,kw) AND ('exercise'/exp OR 'kinesiotherapy'/exp OR 'fitness'/exp OR 'sport'/exp OR 'exercise*':ti,ab,kw OR 'physical activit*':ti,ab,kw OR 'aerobic*':ti,ab,kw OR 'physical train*':ti,ab,kw OR 'fitness':ti,ab,kw OR 'sport*':ti,ab,kw) AND ('biological marker'/exp OR 'biomarker*':ti,ab,kw OR 'marker*':ti,ab,kw OR 'blood level'/exp OR 'serum':ti,ab,kw OR 'molecular probe'/exp OR 'probe':ti,ab,kw OR 'probes':ti,ab,kw OR 'genetics'/exp OR 'phenotype'/exp OR 'phenotyp*':ti,ab,kw OR 'endophenotyp*':ti,ab,kw OR 'genet*':ti,ab,kw OR 'gene expression'/exp OR 'gene expression*':ti,ab,kw OR 'transcript*':ti,ab,kw OR 'methylation'/exp OR 'methyl*':ti,ab,kw OR 'epigenetics'/exp OR 'genetic epigenesis'/exp OR 'epigen*':ti,ab,kw OR 'RNA'/exp OR 'rna':ti,ab,kw OR 'proteome'/exp OR 'proteomics'/exp OR 'proteom*':ti,ab,kw OR 'metabolism'/exp OR 'metabol*':ti,ab,kw OR 'biological':ti,ab,kw OR 'neurobiological':ti,ab,kw OR 'molecular mechanism*':ti,ab,kw OR 'cytokine'/exp OR 'cytokin*':ti,ab,kw OR 'interleuk*':ti,ab,kw OR 'inflammat*':ti,ab,kw OR 'oxidative stress':ti,ab,kw OR 'brain derived neurotrophic factor'/exp OR 'bdnf':ti,ab,kw OR 'brain derived neurotrophic factor':ti,ab,kw OR 'neurotrophin*':ti,ab,kw OR 'kynurenine'/exp OR 'kynurenine':ti,ab,kw OR 'tryptophan'/exp OR 'tryptophan':ti,ab,kw OR 'reactive oxygen metabolite'/exp OR 'reactive oxygen species':ti,ab,kw OR 'sex hormone*':ti,ab,kw OR 'testosterone'/exp OR 'testosterone':ti,ab,kw OR 'estrogen'/exp OR 'estrogen*':ti,ab,kw OR 'hydrocortisone'/exp OR 'cortisol':ti,ab,kw OR 'biogenic amine'/exp OR 'monoamine*':ti,ab,kw OR 'serotonin':ti,ab,kw OR 'dopamine':ti,ab,kw OR 'melatonin'/exp OR 'melatonin':ti,ab,kw OR 'noradrenaline':ti,ab,kw OR 'endocannabinoid'/exp OR 'endocannabinoid*':ti,ab,kw OR 'endo-cannabinoid*':ti,ab,kw OR 'endocanabinoid*':ti,ab,kw OR 'anandamide'/exp OR 'anandamide':ti,ab,kw OR '2 arachidonoylglycerol'/exp OR '2-arachidonoylglycerol':ti,ab,kw OR '2-arachidonoyl-glycerol':ti,ab,kw OR '2-arachidonyl-glycerol':ti,ab,kw OR '2-arachidonylglycerol':ti,ab,kw) AND ('clinical trial'/exp OR 'trial':ti,ab,kw OR 'trials':ti,ab,kw OR 'random*':ti,ab,kw OR 'randomization'/exp OR 'control group'/exp OR 'group':ti,ab,kw OR 'groups':ti,ab,kw OR 'controls':ti,ab,kw OR 'double blind procedure'/exp OR 'single blind procedure'/exp OR 'blind*':ti,ab,kw OR 'placebo':ti,ab,kw OR 'rct':ti,ab,kw OR 'rcts':ti,ab,kw OR 'cct':ti,ab,kw OR 'ccts':ti,ab,kw OR 'nct0*':ti,ab,kw,cn OR 'nct1*':ti,ab,kw,cn OR 'nct2*':ti,ab,kw,cn OR 'nct3*':ti,ab,kw,cn OR 'nct4*':ti,ab,kw,cn OR 'nct5*':ti,ab,kw,cn OR 'nct6*':ti,ab,kw,cn OR 'nct7*':ti,ab,kw,cn OR 'nct8*':ti,ab,kw,cn OR 'nct9*':ti,ab,kw,cn) NOT 'conference abstract'/it

7081 results on May 6, 2024

**Cochrane Central Register of Controlled Trials (CENTRAL)**

#1 MeSH descriptor: [Depression] explode all trees

#2 MeSH descriptor: [Depressive Disorder, Major] explode all trees

#3 MeSH descriptor: [Antidepressive Agents] explode all trees

#4 (depress* OR (depressive NEXT dis*) OR (depressive NEXT symptom*) OR antidepress* OR

MDD):ti,ab,kw

#5 #1 OR #2 OR #3 OR #4

#6 MeSH descriptor: [Exercise] explode all trees

#7 MeSH descriptor: [Exercise Therapy] explode all trees

#8 MeSH descriptor: [Physical Fitness] explode all trees

#9 MeSH descriptor: [Sports] explode all trees

#10 (exercise* OR (physical NEXT activit*) OR aerobic* OR (physical NEXT train*) OR fitness OR

sport*):ti,ab,kw

#11 #6 OR #7 OR #8 OR #9 OR #10

#12 MeSH descriptor: [Biomarkers] explode all trees

#13 MeSH descriptor: [] explode all trees and with qualifier(s): [blood - BL]

#14 MeSH descriptor: [Molecular Probes] explode all trees

#15 MeSH descriptor: [] explode all trees and with qualifier(s): [genetics - GE]

#16 MeSH descriptor: [Phenotype] explode all trees

#17 MeSH descriptor: [Gene Expression] explode all trees

#18 MeSH descriptor: [Methylation] explode all trees

#19 MeSH descriptor: [Epigenomics] explode all trees

#20 MeSH descriptor: [Epigenesis, Genetic] explode all trees

#21 MeSH descriptor: [RNA] explode all trees

#22 MeSH descriptor: [Proteome] explode all trees

#23 MeSH descriptor: [Proteome] explode all trees

#24 MeSH descriptor: [Metabolism] explode all trees

#25 MeSH descriptor: [] explode all trees and with qualifier(s): [metabolism - ME]

#26 MeSH descriptor: [Cytokines] explode all trees

#27 MeSH descriptor: [Brain-Derived Neurotrophic Factor] explode all trees

#28 MeSH descriptor: [Kynurenine] explode all trees

#29 MeSH descriptor: [Tryptophan] explode all trees

#30 MeSH descriptor: [Reactive Oxygen Species] explode all trees

#31 MeSH descriptor: [Testosterone] explode all trees

#32 MeSH descriptor: [Estrogens] explode all trees

#33 MeSH descriptor: [Hydrocortisone] explode all trees

#34 MeSH descriptor: [Biogenic Monoamines] explode all trees

#35 MeSH descriptor: [Melatonin] explode all trees

#36 MeSH descriptor: [Endocannabinoids] explode all trees

#37 (biomarker* OR marker* OR serum OR probe OR probes OR phenotyp* OR endophenotyp* OR genet*

OR (gene NEXT expression*) OR transcript* OR methyl* OR epigen* OR RNA OR proteom* OR

metabol* OR biological OR neurobiological OR (molecular NEXT mechanism*)):ti,ab,kw

#38 (biomarker* OR marker* OR serum OR probe OR probes OR phenotyp* OR endophenotyp* OR genet*

OR (gene NEXT expression*) OR transcript* OR methyl* OR epigen* OR RNA OR proteom* OR

metabol* OR biological OR neurobiological OR (molecular NEXT mechanism*) OR cytokin* OR

interleuk* OR inflammat* OR "oxidative stress" OR BDNF OR "brain derived neurotrophic factor" OR

neurotrophin* OR kynurenine OR tryptophan OR "reactive oxygen species" OR (sex NEXT hormone*)

OR testosterone OR estrogen* OR cortisol OR monoamine* OR serotonin OR dopamine OR melatonin

OR noradrenaline OR endocannabinoid* OR endo-cannabinoid* OR endocanabinoid* OR anandamide

OR "2-arachidonoylglycerol" OR "2-arachidonoyl-glycerol" OR "2-arachidonyl-glycerol" OR "2-

arachidonylglycerol"):ti,ab,kw

#39 #12 OR #13 OR #14 OR #15 OR #16 OR #17 OR #18 OR #19 OR #20 OR #21 OR #22 OR #23 OR #24 OR

#25 OR #26 OR #27 OR #28 OR #29 OR #30 OR #31 OR #32 OR #33 OR #34 OR #35 OR #36 OR #37 OR

#38

#40 #5 AND #11 AND #39

3661 results on May 6, 2024
